# Evidence that collaborative action between local health departments and nonprofit hospitals helps foster healthy behaviors in communities: a multilevel study

**DOI:** 10.1186/s12913-020-05996-8

**Published:** 2021-01-02

**Authors:** Geri Rosen Cramer, Gary J. Young, Simone Singh, Jean McGuire, Daniel Kim

**Affiliations:** 1 Bouvé College of Health Sciences and the Center for Health Policy and Healthcare Research, Northeastern University, Boston, MA USA; 2 Bouvé College of Health Sciences, D’Amore-McKim School of Business and the Center for Health Policy Healthcare Research, Northeastern University, Boston, MA USA; 3 Department of Health Management and Policy, University of Michigan, Ann Arbor, USA; 4 Bouvé College of Health Sciences, Northeastern University, Boston, MA USA; 5 Bouvé College of Health Sciences and the School of Public Policy and Urban Affairs, Northeastern University, Boston, MA USA

**Keywords:** Collaboration, Population health, Local health department, Nonprofit hospital, Social capital, Health behaviors

## Abstract

**Background:**

The Patient Protection and Affordable Care Act of 2010 (ACA) encouraged nonprofit hospitals to collaborate with local public health experts in the conduct of community health needs assessments (CHNAs) for the larger goal of improving community health. Yet, little is known about whether collaborations between local health departments and hospitals may be beneficial to community health. In this study, we investigated whether individuals residing in communities with stronger collaboration between nonprofit hospitals and local public health departments (LHDs) reported healthier behaviors. We further explored whether social capital acts as a moderating factor of these relationships.

**Methods:**

We used multilevel cross-sectional models, controlling for both individual and community-level factors to explore LHD-hospital collaboration (measured in the National Association of County and City Health Officials (NACCHO) Forces of Change Survey), in relation to individual-level health behaviors in 56,826 adults living in 32 metropolitan and micropolitan statistical areas, captured through the 2015 Behavioral Risk Factor Surveillance System (BRFSS) SMART dataset. Nine health behaviors were examined including vigorous exercise, eating fruits and vegetables, smoking and binge drinking. Social capital, measured using an index developed by the Northeast Regional Center for Rural Development, was also explored as an effect modifier of these relationships.

**Results:**

Stronger collaboration between nonprofit hospitals and LHDs was associated with not smoking (odds ratio, OR 1.32, 95% CI 1.11 to 1.58), eating vegetables daily (OR 1.29; 95% CI 1.06 to 1.57), and vigorous exercise (OR 1.17; 95% CI 1.05 to 1.30). The presence of higher social capital also strengthened the relationships between LHD-hospital collaborations and wearing a seatbelt (p for interaction = 0.01) and general exercise (p for interaction = 0.03).

**Conclusions:**

Stronger collaboration between nonprofit hospitals and LHDs was positively associated with healthier individual-level behaviors. Social capital may also play a moderating role in improving individual and population health.

## Background

In 2014, the United States ranked last among high-income countries on measures of mortality and life expectancy while health expenditures per person were more than double the highest-ranking country [1, 2]. Within the US, significant health disparities exist within and across populations and regions [3]. The pioneering work of the Robert Wood Johnson Foundation’s “Culture of Health Action Framework” and the Institute for Healthcare Improvement (IHI)‘s “Triple Aim,” have proposed that health is a function of community assets and socioeconomic circumstances [4, 5]. Cross-sector partnerships between local public health departments and healthcare providers are a potentially important resource for improving community health and wellbeing.

However, a long-standing chasm has existed between the public health and healthcare delivery sectors [6, 7]. In general, local health departments (LHDs) have the mission to promote community health through programs aimed at disease prevention and emergency preparedness. Meanwhile, healthcare providers focus on patient-level treatment for acute and chronic health conditions. Among healthcare providers, hospitals are a particularly important community asset as they possess knowledge, data, and human capital resources that are relevant for improving community health [8]. Although over half of US hospitals are nonprofit, tax-exempt entities and therefore carry societal and governmental expectations to provide community benefits, these hospitals have typically sought to meet this expectation by providing charity care to the poor or investing in health education and research activities [9]. These efforts address important needs but do little to directly improve community health [9]. Collaboration between LHDs and hospitals may be impeded by differences in mission, language and training of those in leadership positions [10].

At the same time, empirical research has explored the potential benefits that can be gained from greater levels of collaboration between LHDs and hospitals. One of the few relevant studies found that when hospitals and LHDs do collaborate on community health needs assessments, the results are more comprehensive and detailed than when either entity (LHD and hospital) conducted an assessment independently [11]. In addition, an ecological study conducted by Mays et al. showed that greater collaboration among various stakeholders was associated with lower county-level mortality rates [12]. Yet, a significant knowledge gap exists in that none of these studies have implemented a multilevel study design to directly test whether collaboration between LHDs and hospitals is associated with health behaviors when measured at the individual-level.

### Conceptual framework

**Fig. 1:**
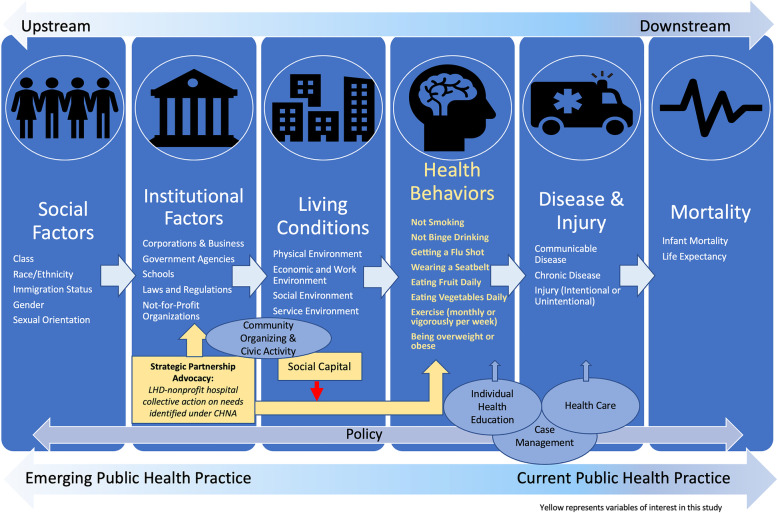
A Modified BARHII Public Health Framework for Reducing Health Inequities [13] (adapted)

Although there are many relevant frameworks in the public health, sociology and economics literature that address community health and welfare, we adapt a framework created by the Bay Area Regional Health Inequities Initiative (BARHII, modified framework in Fig. 1) [13].

The BARHII Framework for Reducing Health Inequities has become a useful tool for LHDs seeking to better address social determinants [13]. This framework is particularly suitable for our work in that it recognizes the importance of strategic partnerships between institutions and articulates that individual health behaviors are on the path to individual disease and mortality.

One area of interest not explicitly identified in the BAHRII Framework is the concept of social capital. In a community context, social capital has been defined as the “source of the ability to identify problems and needs, achieve a workable consensus on goals and priorities, and work in partnership with others to achieve goals.” [14, 15] Putnam, in his seminal work “Bowling Alone”, described community-level social capital as being a “public good” whereby efforts from collective action may benefit the population at large [16].

In this study, we sought to address two key research questions using a multilevel study design: (1) are individuals residing in communities with long-standing LHD-hospital collaborative action on community-wide health promotion more likely to report healthier behaviors than individuals residing in communities with less collaborative action?; and (2) does the level of social capital of a community modify the relationships between long-standing LHD-hospital collaborative action and health behaviors of individuals?

We hypothesized that individuals residing in communities with long-standing collaborative action would report healthier behaviors. We believe that LHDs and nonprofit hospitals that engage in long-standing collaborative action benefit from the synergies that have been observed through research on collaboration outside of the health context [17]. We also hypothesized that higher levels of community social capital could serve as a facilitator in these efforts. That is, social capital may act as a catalyst for such collaborative action to be effective.

## Methods

### Study population

Our study population consisted of 56,826 adults living in 32 metropolitan and micropolitan statistical areas (MMSA) in the United States. Geographies were selected for which we had individual-level responses and a single LHD that reported the presence of at least one nonprofit hospital within its jurisdiction.

### Outcomes

Information on individual-level health behaviors came from the 2015 Behavioral Risk Factor Surveillance Survey (BRFSS) SMART dataset. The 2015 BRFSS SMART dataset is a sub-sample of the 2015 state BRFSS surveys based on geographies defined as metropolitan statistical areas, micropolitan statistical areas, and metropolitan divisions (collectively called MMSAs) made publicly available to researchers. The 2015 BRFSS Smart dataset included 132 MMSAs where at least 500 BRFSS surveys were collected [18].

Individual health behaviors selected for this study were those identified by the Centers for Disease Control and Prevention (CDC) as “Winnable Battles”. Winnable Battles are health outcomes for which the CDC believes that public health can make significant progress in a relatively short time frame (i.e., within one to 4 years), have a large-scale public impact, and have evidenced-based interventions readily available for ease of implementation. From the “Winnable Battles” list, we included the following modifiable behaviors/conditions into our analysis: smoking, wearing a seatbelt, binge drinking, eating vegetables daily, eating fruit daily, general exercise in a month, vigorous exercise (300 min) in a week and being overweight or obese (based on self-reported height and weight). Additionally, while not designated by the CDC as a “Winnable Battle”, flu vaccinations are also an important measure of community health where LHDs and nonprofit hospitals may collaborate and was thus included in our analysis. While we did not have access to specific collaborative action strategies, many of the included measures are commonly identified in community health needs assessments (CHNAs) as being health needs in the community (healthy eating, physical activity, smoking, etc.) [19, 20].

In addition to studying the impact of LHD-hospital collaboration on specific individual health behaviors, we also conducted analyses using two index measures of the health behaviors. We created one index for risky behaviors and another for healthy lifestyle behaviors (healthy eating and exercise).

The risky behaviors index included wearing a seatbelt, not smoking, not binge drinking and getting a flu shot. For each individual respondent, we assigned a score for this index based on the number of specific behaviors that the individual reported undertaking (or in the case of smoking and binge drinking reported not undertaking). Thus, if an individual did not report undertaking any of these behaviors, we assigned a score of zero. If an individual reported all of the behaviors, we assigned a score of four. To create the healthy lifestyle index, we combined the specific behaviors of eating vegetable(s) daily, eating fruit daily and vigorously exercising. We found a high correlation between general exercise and vigorous exercise and felt the latter was more representative of a healthy lifestyle, so we included that variable only. For this index, we also assigned a score to each individual respondent based on the number of behaviors that reportedly were undertaken. Thus, the healthy lifestyle index ranged from zero (if an individual did not report any of the variables) to three (if an individual reported eating fruit, vegetables and vigorously exercising). Index variables were analyzed as continuous outcomes.

### Predictor variables

The data for assessing the level of collaboration between LHDs and nonprofit hospitals came from the 2015 Forces of Change Survey administered by the National Association of County and City Health Officials (NACCHO). This survey was developed to measure the impacts of economic forces on the budget, staff, and programs of LHDs. The survey was administered to a subset of the nearly 3000 LHDs across the country using stratified random sampling (on state and size of population in the LHD jurisdiction) [21]. Nine hundred and forty-eight (948) LHDs were randomly selected, of which 690 LHDs participated (73% response rate). Approximately 77% of the included LHDs reported having at least one nonprofit hospital in their jurisdiction (*n* = 519).

We used each LHD’s response to a single question as a proxy for whether it had a long-standing collaboration with nonprofit hospitals in the community for which the LHD was responsible [22, 23]. The survey question asks “Is your LHD included in any nonprofit hospital’s implementation plan for the community health needs assessment (CHNA)?” Response options included: no collaboration, participating in the development of a hospital implementation plan, listed as a partner in a hospital implementation plan, conducting an activity together in a hospital implementation plan, and using the same implementation plan as the hospital. Because we were interested in identifying established collaborations between LHDs and hospitals within local communities, we created a binary variable indicating “long-standing collaboration” for those LHDs that reported conducting an activity together or using the same implementation plan as the nonprofit hospital in their community. Although the survey question did not specify a defined time period for reported LHD-hospital collaboration, such CHNA implementation efforts typically entail multiple years of activity. Accordingly, we interpreted LHD responses indicating a joint effort for implementing community health needs assessments to be reflective of relatively long-standing relationships (or lack thereof) between an LHD and one or more nonprofit hospitals in a community. While this variable lacked granularity in terms of the nature, strength, and scale of LHD-hospital collaboration (e.g., the content of implementation plans was not known), previous research suggests that any level of meaningful, ongoing collaboration between these two sectors within the same community is uncommon [24]. Thus, we constructed this variable to measure if such collaboration is associated with positive individual-level health outcomes.

### Social capital as a potential effect modifier

To assess whether community social capital moderated the relationship between LHD-hospital collaboration and individual-level health behavior, we used the 2014 Northeast Regional Center for Rural Development (NRCRD) social capital index as developed by Rupasingha et al. (2006) [25]. This social capital index was created using principal component analysis based on four factors: the percentage of voters who voted in presidential elections, the response rate to the Census Bureau’s decennial census, the number of non-profit organizations, and the number of social organizations and associations [25]. For social capital, the geographic unit was the county in which the LHD was located. While this was not the unit for the primary analysis, a metropolitan or micropolitan statistical area (MMSA) is defined as a region that consists of a city and surrounding communities that are linked by social and economic factors, as established by the U.S. Office of Management and Budget (OMB) [18]. Thus, social capital at the county-level was deemed an adequate measure for this analysis.

### Covariates

In all models, we controlled for factors at the individual level (age, marital status, race, education, household income, and health insurance status from the BRFSS, all as categorical variables except for age) and the community-level (median household income and percent black), derived from the 2010 U.S. Census and Area Resource File [26–28]. Individual survey responses were linked to community-level characteristics from the NRCRD social capital index and NACCHO Profile Survey (2013). We included a variable indicating whether an LHD had more than the average number of full-time equivalents (FTEs) from all LHDs responding to the 2013 NACCHO Profile survey. FTEs relate to a LHD’s resource availability that may affect collaborative action as well as health outcomes [29]. We also controlled for the presence/absence of a local board of health, which has been shown to influence collaboration between nonprofit hospitals and LHDs [23]. Finally, we controlled for whether the state had expanded Medicaid as of 2015.

### Statistical analysis

Due to missing data for two covariates—whether the individual identified as being insured and the individual’s income (35 and 18%, respectively)—we implemented a multiple imputation approach using proc. mi, SAS version 9.4 [30]. To assess whether there was residual confounding in missing observations for the health insurance variable, we conducted a sensitivity analysis that excluded the health insurance variable from our models that analyzed the two health behavior indices. Missing data on the outcomes of interest (ranging from 4.3% for not smoking to 13.2% for vigorous exercise) was cause for excluding the individual from the analysis.

To examine the relationship between LHD-hospital collaboration and an individual’s reported health behavior, we estimated hierarchical generalized linear models using proc. glimmix [31]. We generalized the individual-level logistic regression model for binary and continuous outcomes and incorporated the aforementioned community- and individual-level covariates. All models incorporated random intercepts to capture the variation among communities and to adjust the estimates for the lack of independence and clustering of individual responses within each community. Hypothesis testing was two-sided with a type 1 error rate of 0.05.

## Results

As noted, after data linkages our sample comprised 56,826 respondents representing 32 communities across 25 states. Geographies were excluded if there was no data from both the Forces of Change survey or BRFSS Smart and if the MMSA (from the BRFSS Smart) represented more than one LHD. Table 1 compares descriptive statistics between respondents from these 32 communities and those from excluded communities. The two groups were largely comparable except for race/ethnicity, whereby included communities’ members were more likely to self-identify as white (83.3% vs 79.6%).

**Table 1:** Comparison of characteristics of individuals living in included vs. excluded communities in full BRFSS Smart cohort (total of 130 MMSAs)

	Included communities (***n*** = 32 MMSAs); % of all respondents	Excluded communities (***n*** = 98 MMSAs); % of all respondents
Sex		
Male	48.7	48.3
Female	51.3	51.7
Marital		
Married	51.4	49.3
Divorced/Widowed/Separated	19.9	19.2
Never Married	23.6	25.6
Member unmarried couple	4.4	5.1
Refused	0.6	0.8
Education		
Less than Grade 12	12.6	13.3
Grade 12 or GED (High school graduate)	26.8	25.4
College 1 year to 3 years (Some college or technical school)	32.0	30.0
College 4 years or more (College graduate)	28.2	30.8
Refused	0.4	0.5
Employment Status		
Employed	49.9	51.0
Not employed	13.6	13.0
Retired	29.2	28.8
Unable to work	6.3	6.3
Refused	1.0	0.9
Income		
Less than $20,000	12.3	13.3
$20,000 to $75,000	41.7	38.7
Greater than $75,000	28.6	30.9
Refused/Don’t know	17.3	17.2
Preferred Race		
White	83.3	79.6
Black or African American	8.1	11.1
Asian	1.9	2.9
Other	4.3	4.2
Refused/Don’t know	2.5	2.2
Age		
Under 30	11.0	10.5
30–50	25.1	24.7
50–65	29.5	30.4
Over 65	33.1	33.0
Refused/Don’t Know/Missing	1.3	1.4

Within our sample, approximately 14% of the population reported smoking, 12% reported binge drinking, 11% reported not wearing a seatbelt and 51% reported not getting a flu shot. Despite most of the individuals reporting that they ate fruits and vegetables daily (62 and 80% respectively), 65% of respondents identified as being overweight or obese. Seventy-five percent (75%) of individuals reported that they performed some type of exercise monthly but only 38% reported vigorously exercising weekly.

Variation also existed for the key independent variable, the level of LHD-hospital collaboration. Approximately 22% of the communities in our sample reported long-standing collaboration between the LHD and one or more hospitals in its community (7 of 32 LHDs). Thus, 78% of the communities in our sample reported relatively little or no LHD-hospital collaboration. Those LHDs answering “I don’t know” were assumed to have no collaboration with nonprofit hospitals, an assumption previously used in the peer-reviewed literature [23]. Thus, in line with previous studies, long-standing collaboration between LHDs and hospitals appeared to be uncommon and thus, the binary variable for LHD-hospital collaboration included in the present study appears to capture an important qualitative distinction among the 32 communities represented in our study sample.

From multilevel analyses where both individual and community characteristics were controlled, we found significant and positive associations between LHD-hospital collaboration and the composite indices of risky behaviors (OR = 1.18; 95% CI = 1.10–1.28; Table 2) and healthy nutrition/lifestyle behaviors (OR = 1.12; 95% CI = 1.05–1.19; Table 3). LHD-hospital collaboration was also significantly and positively associated with three individual outcomes: not smoking (OR = 1.32, 95% CI = 1.11–1.58; Table 2), eating vegetables daily (OR = 1.29; 95% CI = 1.06–1.57; Table 3) and vigorous exercise (OR = 1.17; 95% CI = 1.05–1.30; Table 3). In sensitivity analyses, removing the health insurance variable from the models did not materially change our findings.

**Table 2:** Unstratified Odds Ratios of Individual-Level Risky Behaviors

	Wearing a Seatbelt		Not Smoking		Not Binge Drinking		Getting a Flu Shot		Risky Behavior Index	
	Odds Ratio	95% CI	Odds Ratio	95% CI	Odds Ratio	95% CI	Odds Ratio	95% CI	Odds Ratio	95% CI
*Primary Predictors of Interest*										
Collaborative action	1.55	0.96, 2.49	1.32*	1.11, 1.58	1.19	0.92, 1.55	1.03	0.89, 1.20	1.18*	1.10, 1.28
Social capital index	1.09	0.67, 1.76	0.94	0.80, 1.10	0.93	0.75, 1.16	1.41*	1.07, 1.87	1.15*	1.05, 1.25
*Individual-level Covariates*										
Male	0.49*	0.41, 0.58	0.63*	0.48, 0.82	0.47*	0.39, 0.57	0.79*	0.69, 0.91	0.76*	0.69, 0.83
Married	1.48*	1.12, 1.95	1.64*	1.31, 2.05	1.35*	1.00, 1.83	1.13*	1.01, 1.27	1.23*	1.09, 1.38
College	1.37*	1.09, 1.72	1.37*	1.17, 1.61	0.94	0.70, 1.24	1.25*	1.07, 1.46	1.17*	1.07, 1.27
Black	0.86	0.57, 1.30	1.02	0.72, 1.42	1.45*	1.06, 1.98	0.81*	0.69, 0.95	0.89	0.71, 1.12
Hispanic	1.10	0.76, 1.58	1.56*	1.13, 2.15	1.16	0.72, 1.87	0.87	0.68, 1.11	0.90*	0.85, 0.95
Asian	1.05	0.68, 1.64	3.10*	1.05, 9.16	3.08*	1.50, 6.32	1.10	0.77, 1.55	1.04	0.86, 1.25
Other Race	1.05	0.43, 2.57	0.99	.042, 2.32	0.97	0.49, 1.90	0.97	0.48, 1.99	1.01	0.76, 1.32
Age	1.16*	1.11, 1.22	1.09*	1.01, 1.17	1.35*	1.24, 1.48	1.36*	1.29, 1.43	1.14*	1.11, 1.17
Insured	1.22	0.75, 1.97	1.64*	1.31, 2.04	1.06	0.85, 1.31	1.98*	1.46, 2.67	1.28*	1.15, 1.43
Income < $15 k	0.94	0.47, 1.92	0.45*	0.33, 0.62	1.19	0.86, 1.65	0.88	0.64, 1.21	0.93	0.74, 1.17
Income $15 k–75 k	0.88	0.66, 1.19	0.68*	0.54, 0.86	1.13	0.95, 1.36	0.93	0.81, 1.07	0.97	0.88, 1.06
*Community-level Covariates*										
Median household income	1.04	0.93, 1.16	1.03	0.97, 1.11	0.95	0.90, 1.01	1.07	0.96, 1.18	1.00	0.97, 1.04
Percent Black	1.03	0.78, 1.36	0.93	0.83, 1.04	0.98	0.85, 1.12	0.91	0.82, 1.02	0.98	0.94, 1.02
Above Average FTEs	0.99	0.64, 1.55	1.30*	1.09, 1.56	0.82	0.61, 1.12	0.76	0.56, 1.04	0.93	0.81, 1.07
Local Board of Health	0.71	0.49, 1.02	0.92	0.74, 1.13	1.11	0.83, 1.65	0.90	0.67, 1.23	0.92	0.83, 1.01
*State-level Covariates*										
Expanded Medicaid	0.72	0.49, 1.08	0.86	0.74, 1.02	0.79*	0.63, 0.98	0.78*	0.67, 0.91	0.86*	0.80, 0.93
*p-value for interaction*	0.01		0.46		0.17		0.88		0.91	

*P < 0.05. The social capital index was dichotomized into communities with an index above the median (1 = yes) and below the median in our sample (0 = no). Individual-level covariates included being male, being married, graduating college, being Black, Asian, Hispanic, or Other race, having income in the specified range (<$15,000/year, 15,000 to 75,000/year), and having health insurance. The reference category for education was having less than a college degree and the reference for income was greater than $75,000 per year. The remaining dichotomous predictors were coded as 1 = yes; 0 = no. The number of FTEs at the LHD was =1 if the LHD had more than the average FTEs in the Profile 2013 survey (mean = 65). Community-level covariates included continuous measures of the population: percent black (Area Resource File, 2016; rescaled to a one-standard deviation change) and median household income (2014; rescaled to a $10,000 unit change). A second model with an interaction term between collaborative action and social capital was also analyzed. The *p*-value for interaction is provided.

**Table 3:** Unstratified Odds Ratios of Individual-Level Healthy Lifestyle Behaviors

	Eating Vegetables		Eating Fruit		General Exercise		Vigorous Exercise		Healthy Weight		Healthy Lifestyle Index	
	Odds Ratio	95% CI	Odds Ratio	95% CI	Odds Ratio	95% CI	Odds Ratio	95% CI	Odds Ratio	95% CI	Odds Ratio	95% CI
*Primary Predictors of Interest*												
Collaborative action	1.29*	1.06, 1.57	1.16	0.99, 1.35	1.17	1.00, 1.38	1.17*	1.05, 1.30	1.06	0.93, 1.21	1.12*	1.05, 1.19
Social capital index	1.10	0.94, 1.29	1.11	0.92, 1.33	0.91	0.73, 1.13	1.05	0.92, 1.19	0.95	0.78, 1.17	1.07*	1.01, 1.14
*Individual-level Covariates*												
Male	0.64*	0.57, 0.72	0.71*	0.58, 0.87	1.15	0.99, 1.33	1.02	0.92, 1.14	0.44*	0.35, 0.57	0.96	0.88, 1.06
Married	1.35*	1.09, 1.66	1.27*	1.09, 1.48	1.18	0.97, 1.45	1.31*	1.07, 1.62	0.80*	0.71, 0.90	1.12*	1.03, 1.21
College	1.16	0.94, 1.42	1.16*	1.08, 1.25	1.14	0.90, 1.44	1.16*	1.01, 1.34	1.15	0.92, 1.43	1.11*	1.05, 1.18
Black	0.59*	0.42, 0.82	1.17	0.92, 1.49	0.69	0.47, 1.00	0.97	0.81, 1.18	0.52*	0.35, 0.78	0.87	0.74, 1.01
Hispanic	1.13	0.80, 1.60	1.25*	1.07, 1.44	0.76*	0.63, 0.92	0.91	0.74, 1.13	0.59*	0.52, 0.68	0.86*	0.79, 0.94
Asian	0.99	0.72, 1.36	1.28	0.86, 1.90	0.71	0.49, 1.04	0.92	0.68, 1.25	1.68*	1.37, 2.07	0.82*	0.75, 0.89
Other Race	1.07	0.57, 2.01	1.02	0.66, 1.59	1.03	0.51, 2.06	1.08	0.48, 2.45	0.95	0.43, 2.08	1.01	0.81, 1.26
Age	1.03	0.98, 1.08	1.07*	1.04, 1.11	0.85*	0.82, 0.89	0.81*	0.79, 0.83	0.81*	0.77, 0.85	0.99	0.96, 1.01
Insured	1.05	0.78, 1.41	1.08	0.93, 1.26	1.46*	1.03, 2.06	1.28*	1.04, 1.57	1.02	0.81, 1.30	1.11*	1.03, 1.21
Income <$15 k	0.54*	0.34, 0.87	0.70*	0.56, 0.88	0.49*	0.37, 0.64	0.73*	0.55, 0.96	0.92	0.70, 1.22	0.93	0.80, 1.09
Income $15 k–75 k	0.79*	0.64, 0.99	0.91	0.77, 1.08	0.72*	0.62, 0.84	0.86*	0.75, 0.98	0.98	0.83, 1.17	0.97	0.90, 1.03
*Community-level Covariates*												
Median household income	1.06*	1.02, 1.10	1.06	0.98, 1.14	1.02	0.96, 1.08	1.05*	1.01. 1.09	1.02	0.95, 1.10	1.00	0.98, 1.02
Percent Black	0.89*	0.82, 0.97	0.88*	0.78, 1.00	0.94	0.82, 1.08	1.01	0.93, 1.11	0.99	0.89, 1.10	0.99	0.96, 1.03
Above Average FTEs	0.97	0.79, 1.18	1.18	0.91, 1.52	0.99	0.75, 1.31	0.91	0.77, 1.07	1.08	0.85, 1.38	1.01	0.91, 1.11
Local Board of Health	0.82*	0.70, 0.95	0.912	0.75, 1.14	0.99	0.82, 1.19	0.97	0.85, 1.10	1.04	0.84, 1.27	0.96	0.89, 1.03
*State-level Covariates*												
Expanded Medicaid	0.74*	0.65, 0.85	1.10	0.94, 1.29	0.92	0.77, 1.10	0.82*	0.73, 0.93	0.96	0.85, 1.08	0.95	0.90, 1.01
*p-value for interaction*	0.23		0.46		0.03		0.40		0.14		0.13	

*P < 0.05. The social capital index was dichotomized into communities with an index above the median (1 = yes) and below the median in our sample (0 = no). Individual-level covariates included being male, being married, graduating college, being Black, Asian, Hispanic, or Other race, having income in the specified range (<$15,000/year, 15,000 to 75,000/year), and having health insurance. The reference category for education was having less than a college degree and the reference for income was greater than $75,000 per year. The remaining dichotomous predictors were coded as 1 = yes; 0 = no. The number of FTEs at the LHD was =1 if the LHD had more than the average FTEs in the Profile 2013 survey (mean = 65). Community-level covariates included continuous measures of the population: percent black (Area Resource File, 2016; rescaled to a one-standard deviation change) and median household income (2014; rescaled to a $10,000 unit change). A second model with an interaction term between collaborative action and social capital was also analyzed. The *p*-value for interaction is provided.

In models that included the interaction term between LHD-hospital collaboration and social capital, we found a significant, positive interaction for two of our outcome measures: wearing a seatbelt (p for interaction = 0.01; Table 2) and general exercise (p for interaction = 0.03; Table 3). After stratification, collaborative action was not significantly associated with either behavior. There were no interactions for other health behaviors or index variables (Tables 2 and 3).

## Discussion

There is growing recognition that improving population health requires multi-sector collaboration. The Patient Protection and Affordable Care Act of 2010 (ACA) encouraged nonprofit hospitals to collaborate with local public health experts in the conduct of CHNAs for the larger goal of improving community health [32]. Long-standing collaborations between the local public health and hospital sectors, however, appear to be quite limited [10, 11]. A previous study using hospital IRS filings showed that only about half of nonprofit hospitals reported including public health experts in any of their implementation activities [33]. Our findings are less optimistic in that 78% of the LHDs in this analysis reported low to no collaborative action on CHNA implementation with nonprofit hospitals in their community [33, 34].

We set out to study whether collaborative action between LHDs and nonprofit hospitals may impact community health. We found that after controlling for a number of other factors, LHD-hospital collaborative action was significantly associated with several healthy behaviors. Both of the composite health behavior indices showed significant positive associations, indicating that individuals living in communities with some level of LHD-hospital collaboration were more likely to report fewer risky behaviors and a greater number of healthy eating and exercising behaviors than those living in communities without documented long-standing collaborative action. Associations with all measured behaviors were in the hypothesized positive direction and three of these associations were both positive and significant. These findings are in line with the BARHII Framework’s inclusion of strategic partnership at the institutional level. While the exact mechanism by which long-standing collaboration (i.e. alterations in the physical, social or service environment) may manifest its effects are unclear, these data show interesting potential impacts of such strategic collaboration.

For social capital, there was some evidence of moderation for two behaviors: wearing a seatbelt and general exercise. This is consistent with our hypothesis that community social capital resources may allow for a community to benefit from implementation activities put forth through LHD-nonprofit hospital collaborative action. From these data, we believe our inclusion of social capital to the BARHII Framework is warranted; however, further study of the mechanisms by which social capital can benefit collaborative action between hospitals and LHDs would be useful.

Although we report a positive association from LHD-hospital collaborative action and individual-level healthy behaviors, only 22% of the communities represented by study respondents included an LHD that reported long-standing collaboration with nonprofit hospitals. While understanding why such collaboration is uncommon was not the objective of the current study, extant research points to several barriers. First, nonprofit hospitals may choose to focus on strategies that directly impact their current patient market instead of targeting the social and economic barriers to health of residents, especially those that do not receive health care at their institution. This choice minimizes the need to partner with external organizations, including the LHD. Second, funding for action (i.e., implementing programs) may be a concern to hospitals given the long-term nature of many strategies required to improve population health. From the LHD’s perspective, budgets have been challenging, leaving staff to do more with less [6, 8]. Third, while current regulations state that nonprofit hospitals must seek input from public health experts to analyze community needs, there is no such expectation for hospitals when prioritizing and implementing actions to meet those needs [9]. Without state or federal laws that require such efforts, nonprofit hospitals may see no rationale to include LHDs in executing implementation strategies.

### Strengths and limitations

To our knowledge, this study offers the first analysis of long-standing collaborative action between LHDs and hospitals and individual-level health behaviors. Our study is exploratory yet methodologically robust in that we used multilevel models to explore impacts on individual-level behaviors, an advance over previous ecological studies. We believe we are also the first to assess whether social capital may play a role in modifying these relationships. We further focus on multiple modifiable health behaviors identified by the CDC as “winnable battles” that can be pivotal to substantially improving population health across the nation.

Nonetheless, there are several limitations to our study. First, our data was observational in nature, being mostly derived from surveys. While we accounted for community and individual-level covariates, it is possible that factors omitted from our multivariate models could contribute to residual confounding. Relatedly, as a cross-sectional study, we could not rule out reverse causation; due to the emerging nature of this research, 2015 was the first, and only year of available data on collaborative action between LHDs and nonprofit hospitals on CHNA implementation from the perspective of the local health department. However, in line with our conceptual framework, we expect our measure of collaborative action to be indicative of a long-standing LHD-hospital collaboration that has developed over time in the communities studied [34]. Hence, the associations observed in this study represent collaborative relationships between hospitals and LHDs with subsequent community health that are not a direct result of regulation.

Second, because we relied on a single item assessment of long-standing collaboration, it was not possible to assess which specific strategies the LHD and hospital jointly implemented. Health behaviors we measured as outcomes may not directly relate to the actions being undertaken through collaborative action. A qualitative analysis of CHNA implementation reports would be useful to further unpack the elements and strategies of collaboration.

Third, our measure of long-standing collaborative action captures the perspective of the LHD only and may not accurately reflect how hospitals in the community perceived the level of collaborative action on community health improvements activities with the LHD. Data from a prior study assessing collaboration with public health experts from the perspective of the nonprofit hospital showed similarly low levels of collaboration [10, 33].

Finally, while individuals from 25 of the 50 states were included in our sample, our study findings may not necessarily be generalizable to all communities in the US. Future studies that include individuals in the other 25 states would help to expand the external validity of our findings.

## Conclusions

This study assessed whether collaborative action between local health departments and nonprofit hospitals was linked to individual-level self-reported health behaviors. Our findings show positive and significant associations for healthier behaviors (fewer risky behaviors and more healthy lifestyle behaviors). Additional research on collaborative action is needed including program evaluations that focus on health outcomes from specific population health initiatives involving the collaboration of local health departments and hospitals. However, given that many LHDs still do not report any collaborative action with nonprofit hospitals in their area, research is also needed to better understand the barriers to such collaboration. Should our findings be replicated in future work including program evaluations and observational longitudinal studies, they may serve to encourage policy makers to consider incentives for LHDs and hospitals to take collaborative action to impact the health of their communities.

**Fig. 2:**
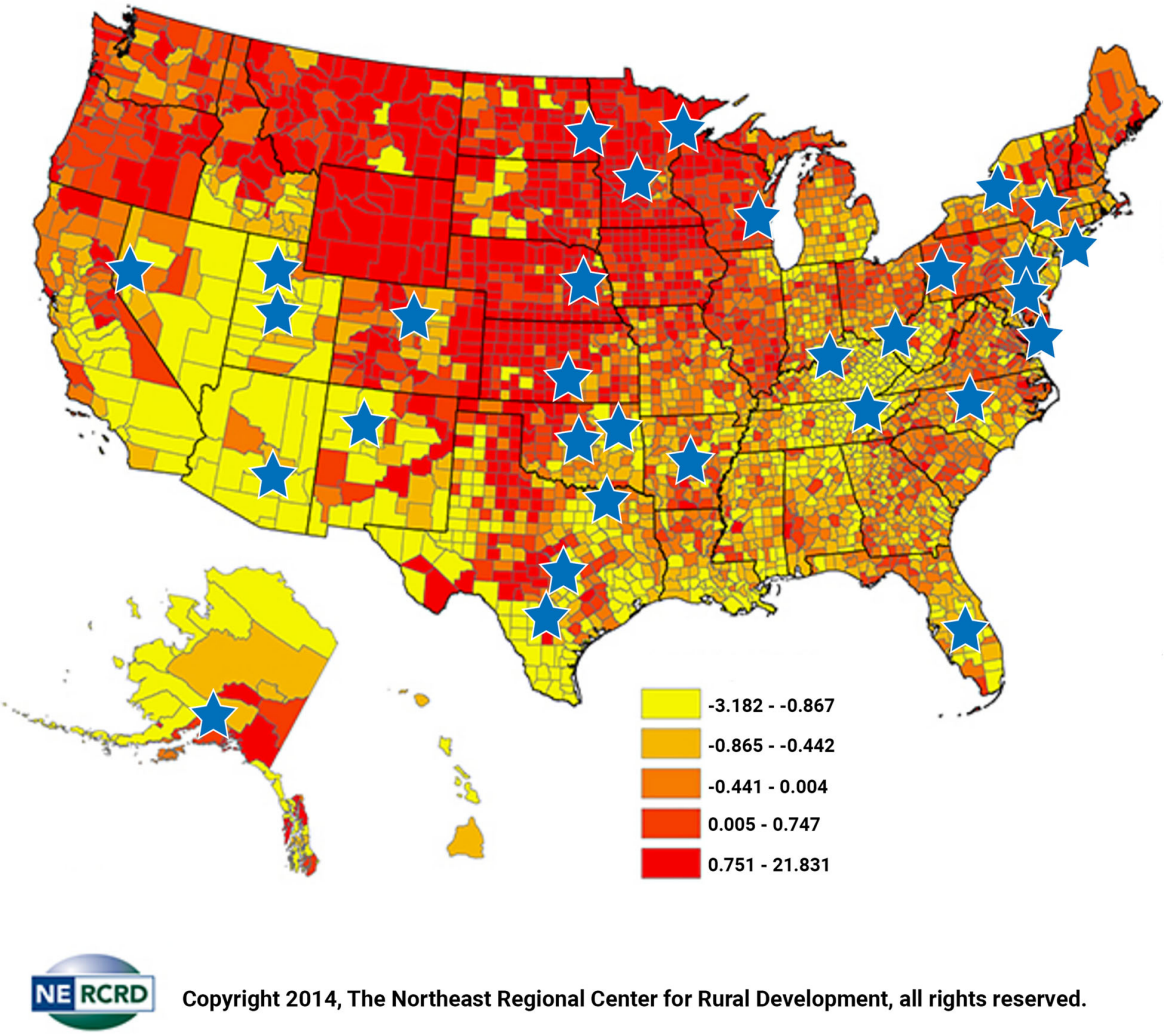
A Map of Included Communities and their Social Capital Index Values [35]**.** Permission was received to use and adapt the copyrighted image

## Data Availability

Data used in this study came from: https://www.cdc.gov/brfss/smart/smart_2015.html (publicly available). https://aese.psu.edu/nercrd/community/social-capital-resources/social-capital-variables-for-2014 (publicly available). http://nacchoprofilestudy.org/forces-of-change/2015-forces-of-change/ (not publicly available). http://nacchoprofilestudy.org/data-requests/ (not publicly available). https://www.hilltopinstitute.org/our-work/hospital-community-benefit/hospital-community-benefit-state-law-profiles/ (publicly available). https://data.census.gov/cedsci/table?d=ACS%205-Year%20Estimates%20Data%20Profiles&table=DP02&tid=ACSDP5Y2015.DP02 (publicly available).

## Abbreviations

ACA: Affordable Care Act
BARHII: Bay Area Regional Health Inequities Initiative
BRFSS: Behavioral Risk Factor Surveillance Survey
CDC: Centers for Disease Control
CHNA: Community Health Needs Assessment
CI: Confidence Interval
FTE: Full-Time Equivalents
IHI: Institute for Healthcare Improvement
LHD: Local Health Department
MMSA: Metropolitan and Micropolitan Statistical Area
NACCHO: National Association of County and City Health Officials
NRCRD: Northeast Regional Center for Rural Development at Penn State
OMB: Office of Management and Budget
OR: Odds Ratio
